# Probing the Hydrogen Enhanced Near-Field Emission of ITO without a Vacuum-Gap

**DOI:** 10.1038/s41598-017-10142-2

**Published:** 2017-08-25

**Authors:** Jacob L. Poole, Yang Yu, Paul R. Ohodnicki

**Affiliations:** 1 National Energy Technology Laboratory, 626 Cochrans Mill Rd, Pittsburgh, PA 15236 USA; 2 AECOM, 626 Cochrans Mill Rd, Pittsburgh, PA 15236 USA

## Abstract

Electromagnetic fields produced by thermal fluctuation can excite the near-field optical states, creating the potential for thermal radiation orders of magnitude greater than what is predicted by Plank’s blackbody theory. The typical schemes employed to probe the trapped electromagnetic energy of the near-field are with considerable technical challenges, suffering from scalability and high costs, hindering widespread use. A waveguide-based scheme relying on photon tunneling is presented as an alternate approach, as waveguides inherently provide a large density of channels for photons to tunnel to with the required k-vector matching and probability density overlap. The conducted experiments with a 10 nm indium tin oxide film, having plasmonic resonance in the 1500 nm wavelength range, show that the near-field EM radiation can be extracted to the far-field by establishing the mode of de-excitation to be that of photon tunneling to a nearby waveguide. Furthermore, it is also demonstrated that the thermally emitted energy is very sensitive to changes in the surface free electron density, a property that is unique to the near-field. In addition to the ease of implementation and scalability, the proposed waveguide-based extraction method does not require a vacuum-gap, which is a significant reduction in the required complexity.

## Introduction

The thermal near-field, capable of generating thermal radiation many orders of magnitude greater than the commonly known far-field, is an active area of research as it is a place where Plank’s theory of blackbody radiation is known to break down, a fact Plank himself was aware of^1–8^. All matter, at temperatures greater than absolute zero, contains thermal energy stored in lattice and electronic vibrational modes, and temperature differentials give rise to the flow of heat, which, in the absence of substantial conductive and convective heat transfer, is in the form of electromagnetic radiation emitted by the thermal fluctuations of charged particles. It is known that surface-plasmon polaritons (delocalized charge-density fluctuations), surface-phonon polaritons (localized charge density fluctuations in polar dielectrics), and surface adsorbate vibrational-modes are mechanisms that can give rise to these enhanced near-field optical density of states^4, 6^. Metal films such as silver and gold are known to have surface-plasmon polariton resonances in the UV-vis wavelength range, whereas silicon carbide and silica are polar materials known to have surface-phonon polariton type resonances at much longer wavelengths^3, 4, 6, 9, 10^. On the other hand, there are a number of doped metal oxides and metal nitrides that exhibit plasmonic resonances in the VIS and NIR frequency ranges^11^.

The thermal near-field is challenging to observe as it is an optical density of states confined to distances much less than the thermal emission wavelength because the substantial majority of the k-vector population is imaginary (non-propagating). To date, complicated techniques such as SNOM and grating assisted coupling were utilized to probe this physics. SNOM consists of bringing coated AFM tips near surfaces to scatter the surface waves to the far-field for observation^3–5, 12^. Whereas, resonant structures such as gratings can also scatter the near-field into the far field, with which the thermally excited surface plasmon resonance of gold and tungsten has been observed^13–15^. In addition, nanoparticles of plasmonic materials such as gold and silver may too scatter the thermal near-field to the far-field through Mie scattering, due to the existence of localized surface plasmon resonance^16–18^. In a recent publication, it was demonstrated that optical fiber type waveguides integrated with functional thin-films can detect emissivity changes upon changing the environment chemistry, an observation from which it surmised that the thermal near-field could be a substantial contributor^19^.

**Figure 1:**
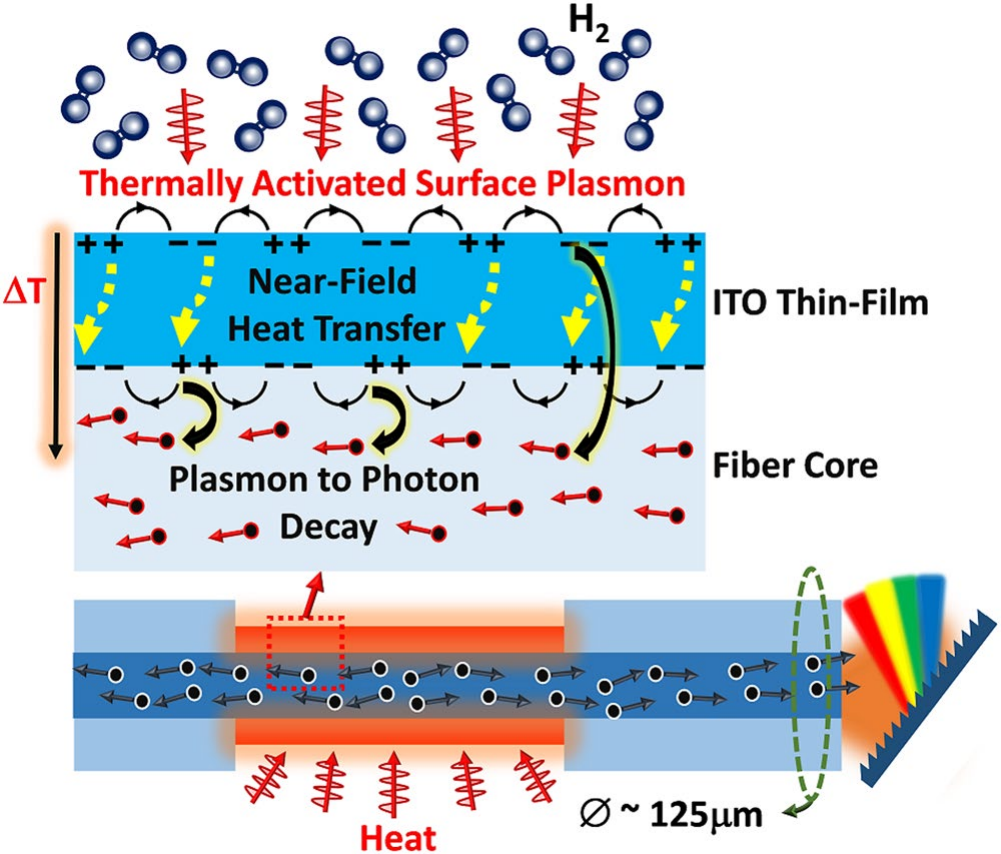
An illustration of the thermal energy stimulated surface plasmon resonance of an indium tin oxide thin-film and the subsequent de-excitation by tunneling to the optical fiber waveguide. The outer surface plasmon couples with the plasmons on the inner side of the film through near-field heat transfer (tunneling, dipole-dipole interactions, etc.).

In this work, we report explicit experimental evidence for the optical waveguide based probing of the thermal near-field of an indium tin oxide (ITO) thin-film, without having to introduce a vacuum-gap (Fig. 1). The ITO film exhibits surface plasmon resonance in the 1500 nm wavelength range contributing a significant near-field density of trapped optical states (imaginary k-vector). In addition, it is shown that the thermal emission is very sensitive to the environmental chemistry induces changes in the surface free electron density, a property which we surmise to be unique to the near-field. The conducted measurements at the plasmon resonance of ITO support the foundation of thermal near-field observation with waveguides, having important applications in thermal energy powered sensors and thermo-photonic energy harvesting, along with providing an inexpensive, scalable, and a practical near-field probing technique^19, 20^. The need for materials that are both high temperature stable and support surface plasmon resonances in the UV-vis and NIR wavelength ranges is clear, given the high value of the possible applications.

### Thermal Energy to Guided Photons

The mechanism is based on the input thermal energy imparting fluctuation to the motions of charged particles, and the subsequent excitation of a charge density wave (Fig. 1) at the outer surface of the film due to the generated electromagnetic fields, which then have several channels to undergo de-excitation through. In addition, a surface charge density wave can be excited at the inner surface of the film, as well. Therefore, the outer surface of the film can transfer heat to the inner surface through tunneling and through dipole-dipole interactions, developing a communication channel via near-field heat transfer, forming a coupled plasmonic molecular state^6^. Which is not unlike the bond formation mechanism between atoms to make molecules. The nearby waveguide supplies a significant number of optical channels, on the order of 10^6^, establishing photon emission by tunneling to be a dominant mode of de-excitation of the surface charge density waves.

### Modeling the Surface Plasmon Resonance

**Figure 2:**
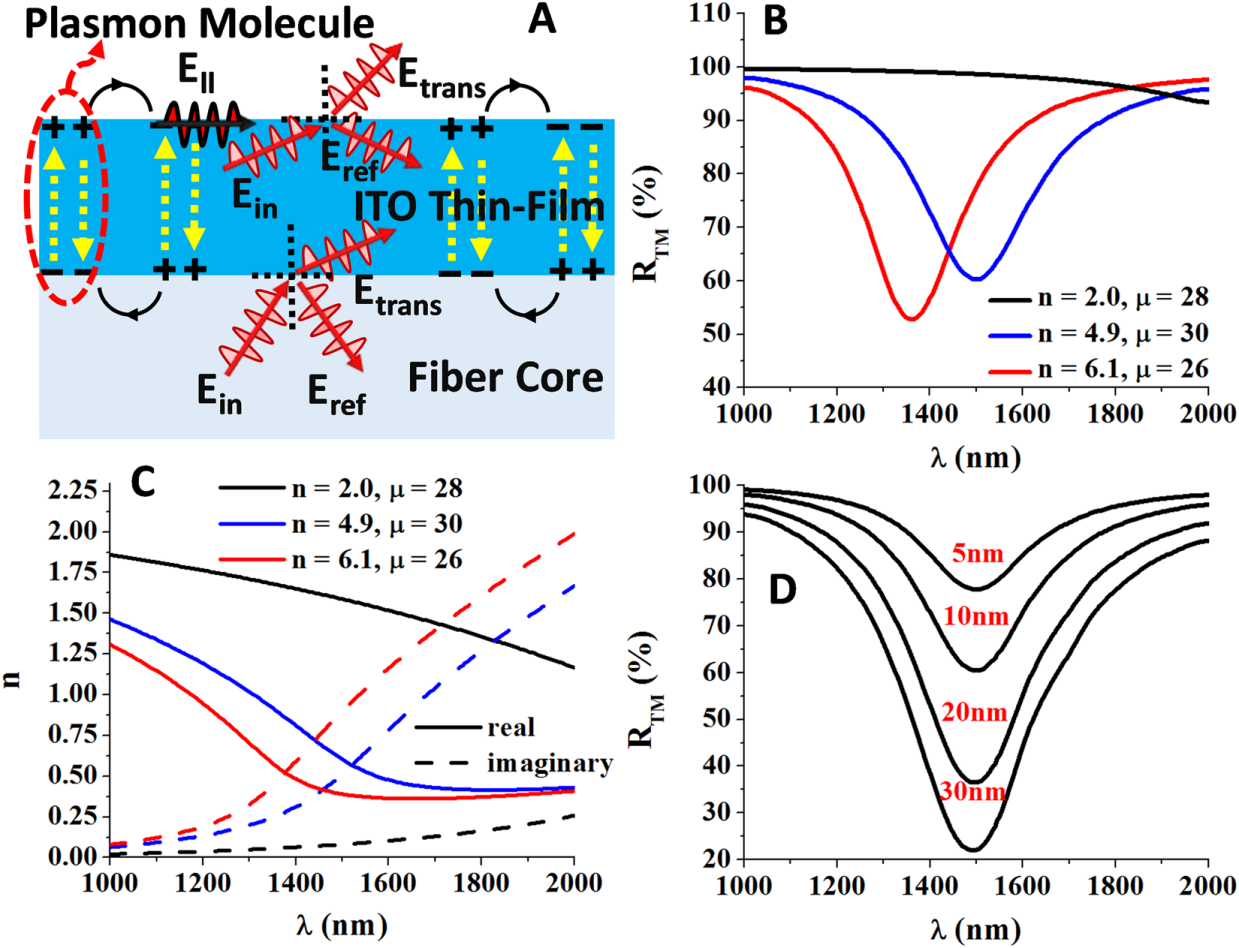
(**A**) Simplified illustration of combining an ITO thin-film with a waveguide without a vacuum-gap. (**B**) Simulation of the transverse magnetic reflectance for a film thickness of 10 nm, and for various carrier concentrations (n in units of 10^20^ cm^−3^) with associated mobility values (µ in units of cm^2^V^−1^s^−1^). (**C**) The real and imaginary parts of the refractive index associated with the carrier concentrations and mobility values. The location of the surface plamon resonance is identified by the intersection of the real and imaginary parts. (**D**) Simulation demonstrating the effect of film-thickness on the magnitude and location of the transverse magnetic reflectance.

The conditions for the existence of an optically excitable surface charge density wave were examined for the illustrated configuration (Figs 1 and 2A) using the transfer matrix approach^21, 22^. Only the transverse magnetic polarization (RTM) has electric field components with surface-parallel k-vectors, needed to stimulate the plasmonic activity. Therefore, only the RTM polarization shows a resonant dip associated with surface plasmon resonance (Fig. 2B), examined at the critical angle inside of the waveguide structure, and located at the intersection of the real and imaginary components of the refractive index (Fig. 2C), where the nominal permittivity values were obtained from Holman *et al*.^23^. With an increase in the carrier concentration from 4.9 to 6.1 × 10^20^ cm^−3^, the resonance peak of the transverse magnetic reflection shifts to lower wavelengths, when the Hall mobility is held relatively constant. At a carrier concentration of 2 × 10^20^ cm^−3^ the plasmon resonance has shifted to a much higher wavelength, outside of the current instrument range, indicating the sensitivity of the location of resonance to this parameter. The dependence of the transverse magnetic reflectance on the film thickness is as shown in Fig. 2D, where an increasing film thickness is accompanied by an increase of loss without altering the location of resonance, which we attribute to be due to an increase in the interaction length.

### Waveguide-Based Near-Field Measurement

**Figure 3:**
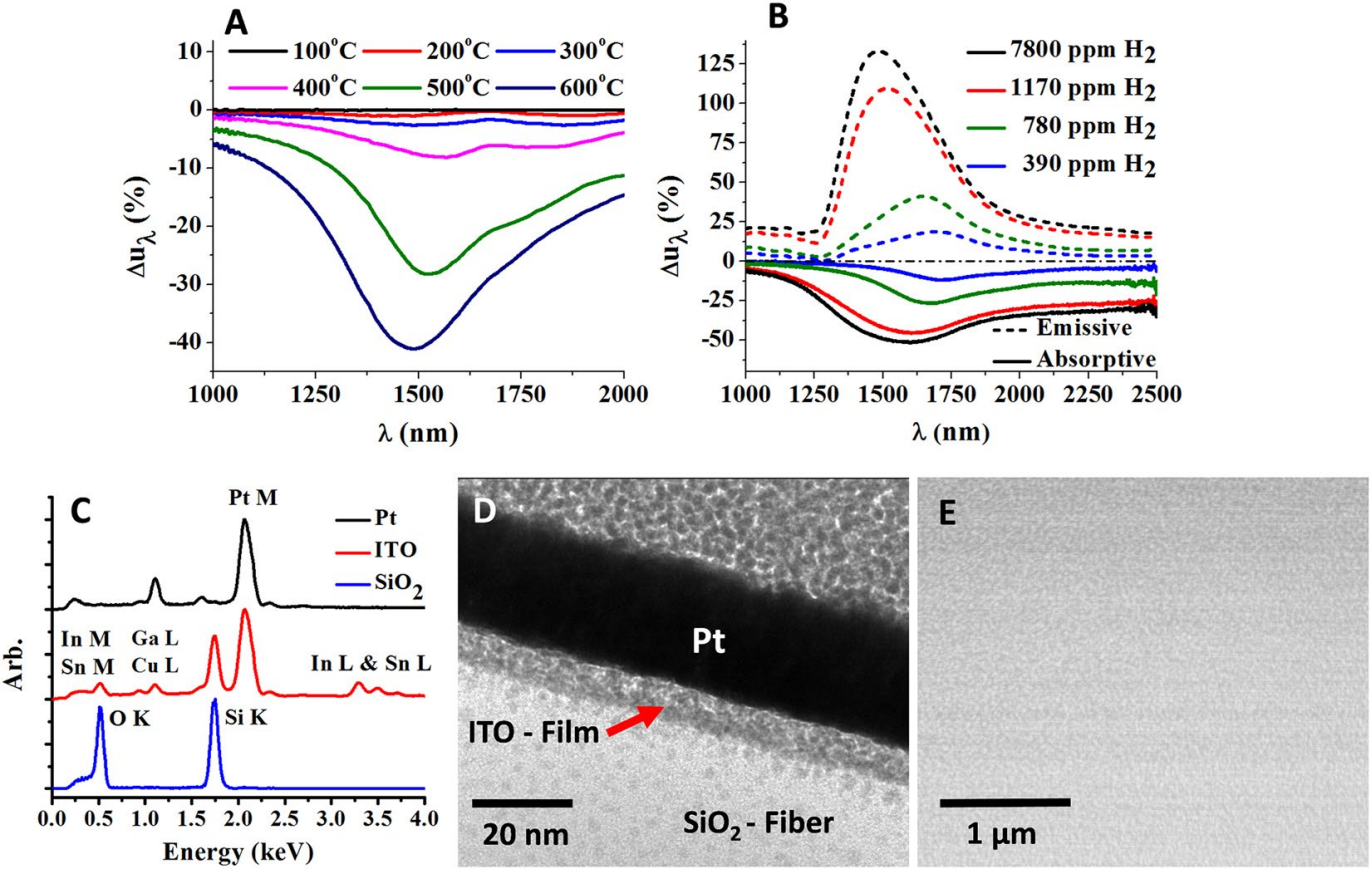
(**A**) Change in the relative spectral energy density Δu_λ_ = (u_λ_ − u_λo_)/u_λo_ upon heating from 300 K to 873 K in N_2_, referenced to 373 K. (**B**) Measured emissive and absorptive near-field optical density of states at plasmon resonance for the 10 nm ITO thin-film at 873 K in various concentrations of H_2_ balanced with N_2_, normalized to 0 ppm of H_2_. The dashed lines correspond to thermal near-field emission based measurements, whereas the solid lines represent measurements conducted with an external light source. (**C**) Cross sectional EDS spot analysis which along with (**D**), the cross sectional TEM image, and (**E**), the BSE SEM image of the surface of a representative ITO film confirm the identity of the film, the thickness of the film to be ~10 nm, and the quality of the film.

The surface plasmon resonance of the ITO thin-film waveguide composite exhibits a shift to shorter wavelengths, in conjunction with a significant increase in absorptivity (Fig. 3A), upon increasing the supplied temperature to 873 K. This is a characteristic of an increase in the free carrier concentration, which is readily concluded from the simulation results presented in Fig. 2B. A clearly observable resonance peak is noted near 1500 nm, confirming that the manufactured film is of high optical quality, along with one that possesses substantial electronic conductivity (~2400Scm^−1^), capable of a sustained surface charge density-wave. When probed with external illumination, successive changes are noted in the plasmon resonance upon exposure to varied concentrations of H_2_ (Fig. 3B - solid line). With an increasing H_2_ partial pressure, a shift of the plasmon resonance to lower wavelengths and an increase in the absorbed light is observed, as expected from simulation. Given that it is challenging to measure absolute absorption in the current configuration, a relative change is measured upon referencing to 1% O_2_ in N_2_, instead, shown as Δu_λ_ in % defined as (u_λ_ − u_λo_)/u_λo_. Referencing to an oxidizing atmosphere allowed for the examination of free carrier concentration mediated changes in the surface plasmons resonance, brought upon by the introduction of an H_2_ partial pressure.

Upon disconnecting the external light source, the near-field thermal radiation can be measured and is shown in Fig. 3B (dashed line). As before, a reference measurement was conducted in a 1% O_2_ background, after which successive increases in the H_2_ partial pressure were introduced. The relative spectral energy density exhibits successive blue shifting as a function of the H_2_ partial pressure, in conjunction with a magnitude change of up to ~125%. This measurement unequivocally shows the observation of the thermal near-field and that the thermal emissivity of the thin-film can be substantially changed by environmental chemistry, which is unique to the near-field, or to k-vectors that are purely imaginary. The observed changes in thermal emission are due to the H_2_ partial pressure mediated changes in the free carrier concentration, evidenced by an increase in the overall flux and a blue shift in the wavelength of the tunneled photons emitted by the de-excitation of the surface charge density waves. In comparing the magnitude of the relative spectral energy density of the thermal emission with that of the absorptive measurement, large deviations are noted. An asymmetry is inherent in the current realization from the perspective of heat flow, and the modifications imparted to the waveguide to integrate the thin-film, which will result in losses due to modal mismatches. However, it is not believed that Kirchoff’s law is violated, which states that absorptivity must equal emissivity, as it is more likely that the asymmetry inherent in the system is the source of the apparent violation. The observed discrepancy could simply be an indicator of the degree of deviation from thermal equilibrium. Lastly, the manufactured film was examined using a transmission electron microscope (TEM) to yield a film thickness measurement of ~10 nm (Fig. 3D), after sectioning with a functional ion beam. The Pt-film was not part of the near-field characterization and its purpose was only to assist with FIB lift-out and the subsequent imaging. An EDS spot scan spectral analysis confirmed the presence of In and Sn in the region representative of the film (Fig. 3C), confirming the identity. The combined images obtained with a TEM and by back scatter electron imaging (Fig. 3E) show the film to be of high optical quality.

## Summary

In this communication, the waveguide-assisted probing of the plasmonic near-field of a 10 nm thick ITO film, is explicitly demonstrated, in the absence of a vacuum-gap and its associated complexities. The waveguide contributes a large density of optical channels with the necessary spatial overlap between the probability densities and k-vector matching to change the preferred mode of de-excitation to photon tunneling, allowing for the extraction of the near-field emission. ITO is a material know to have a substantial electronic conductivity needed to exhibit surface plasmon resonance at NIR frequencies, where a substantial near-field optical density of states with purely imaginary k-vectors exist. The proposed scheme boasts simplicity, ease of implementation, and it is highly cost effective in comparison with the previous employed methods, such as SNOM where an STM-like tip is brought into near-contact with a surface to scatter the near-field into the far-field, or resonant scattering with gratings^3–5, 12–15^. The H_2_ partial pressure induced modulation in the carrier concentration and Hall mobility has a clear effect on the emission spectrum, enhancing the resonant thermally emitted light of the ITO film by 125%. Where, changes in emission due to environmental chemistry are surmised to be unique to the near-field. These observations hold great promise for the development of thermally powered sensors, novel thermal energy harvesting methods, and new instrumentation for probing the thermal near-field optical density of states, all having a geometrical footprint of a human hair.

## Methods

A 105 µm core optical fiber (Thorlabs FGA105-LCA) was processed by etching away the 20 µm silica cladding from a 3 cm section in the center of a 2.5 m segment with buffered hydrofluoric acid (hazardous). The ITO film was deposited onto the exposed fiber core from an alkoxide solution^24–26^ containing InCl_3_, Sn[OCH(CH_3_)_2_]_4_, C_5_H_8_O_2_, and C_3_H_8_O_2_ with a molar ratio of 1:0.065:5.9:28, by dragging a generated droplet at the tip of a 50 µL micro pipette upwards over the exposed core, after which it was calcined in 1%H_2_ at 600 °C inside of a controlled environment tube furnace. The ITO surface plasmon resonance was characterized both by probing with an external light source (Ocean Optics DH-2000-bal), and by observing the thermally emitted light contained in the fiber with an NIR spectrometer (ArcOptics FTNIR-U-09–026). FIB lift-out was conducted with an FEI Nova Nanolab 600 dual beam FIB with a gallium beam, after coating the surface with platinum to maintain film integrity and to assist with film lift-out. Cross-sectional imaging and spot scan EDS analysis was conducted with a 200 kV FEI Tecnai F20 TEM. The back-scattered electron image was obtained with an FEI Quanta 600 F SEM.

The datasets generated during and/or analyzed during the current study are available from the corresponding author on reasonable request.
